# Comparable blood pressure reductions after indoor and outdoor walking exercise

**DOI:** 10.5114/biolsport.2026.156232

**Published:** 2026-01-02

**Authors:** Olga Papale, Emanuel Festino, Francesca Di Rocco, Carl Foster, Iris Prestanti, Sofia Serafini, Pascal Izzicupo, Cristina Cortis, Andrea Fusco

**Affiliations:** 1 Department of Human Sciences, Society and Heath, University of Cassino and Lazio Meridionale, Cassino, 03043, Italy; 2 Department of Exercise and Sport Science, University of Wisconsin-LaCrosse, La Crosse, WI, 54601, USA; 3 Department of Medicine and Aging Sciences, University “G. d’Annunzio” of Chieti-Pescara, Chieti, 66100, Italy

**Keywords:** Cardiovascular diseases, Physical fitness, Post-exercise hypotension, Hiking, Aerobic exercise

## Abstract

Although there is a trend toward outdoor activities, limited research has examined the effects of outdoor exercise on blood pressure. This study aimed to compare the Systolic (sBP) and Diastolic (dBP) Blood Pressure, Heart Rate (HR) response, Post-Exercise Hypotension (PEH) and Rate Pressure Product (RPP) following indoor and outdoor activities. Thirty-seven participants (18 females = age: 24.7 ± 0.7 years, BMI: 21.1 ± 2.1 kg/m^2^, MET*week: 3779 ± 3037; 19 males = age: 24.9 ± 0.5 years, BMI: 24.1 ± 2.6 kg/m^2^, MET*week: 3000 ± 730) completed an outdoor hike (H) (~3800 m) and an indoor maximal walking test (MWT). During both sessions, sBP, dBP and HR were measured 15-min before (PRE), immediately after (POST), 15-min (POST-15) and 30-min (POST-30) after the sessions. Mean differences and standard deviations of all variables, along with PEH and RPP, were determined. Repeated-measures mixed models evaluate the effects of indoor and outdoor sessions on hemodynamic variables. Paired t-tests compared PEH between settings. Regardless of sessions PRE measurements were higher (p < 0.0001) than POST-30 for sBP (H: 10.4 ± 2.1 mmHg; MWT: 12.3 ± 2.3 mmHg), dBP (H: 4.3 ± 1.9 mmHg; MWT: 4.5 ± 1.7 mmHg), HR (H: -5.4 ± 2.2 bpm; MWT: -7.8 ± 2.5 bpm) and RPP (H: 140.6 ± 296.6 mmHg*bpm; MWT: 38.6 ± 323.5 mmHg*bpm). No significant difference in PEH (0.9 ± 11.3 mmHg) was found between sessions. PEH occurred regardless of PRE values, confirming the positive effect of physical activity on reducing BP.

## INTRODUCTION

Physical activity is associated with improved health and quality of life, and even minor improvements in fitness have been linked to reduced cardiovascular and all-cause mortality [1–3]. Current physical activity guidelines recognize the importance of fitness and recommend that adults aged 18–65 years engage in at least 150 minutes per week of moderate-intensity exercise or 75 minutes per week of vigorous-intensity exercise [4]. In addition to those benefits, the World Health Organization (WHO) identifies physical activity as a protective factor for the prevention and treatment of cardiovascular diseases [5]. These conditions include a wide range of disorders affecting the cardiac muscle and vascular system, which supply the heart, brain and other vital organs, and are frequently associated with hypertension. In particular, high blood pressure is recognized as a leading risk factor for global mortality, with more than 10 million attributable deaths annually [1]. Since persistent hypertension, together with the absence of drug or physical therapy over time, may cause irreversible cardiovascular damage, the American Heart Association and the American College of Cardiology have defined blood pressure categories [6]. This classification is expressed as ranges that clarify the clinical implications and risks of hypertension, as shown in Table 1.

**TABLE 1:** Categories and ranges of blood pressure with corresponding health conditions.

Categories	Conditions
Normal: Systolic Blood Pressure < 120 mmHg Diastolic Blood Pressure < 80 mmHg	Optimal cardiovascular health, lower risk of cardiovascular disease

Elevated: Systolic Blood Pressure 120–129 mmHg Diastolic Blood Pressure < 80 mmHg	Elevated risk for the development of hypertension

Hypertension Stage 1: Systolic Blood Pressure 130–139 mmHg Diastolic Blood Pressure 80–89 mmHg	Mild to moderate elevation in blood pressure levels which warrants lifestyle modifications and possibly pharmacological treatment depending on individual risk factors

Hypertension Stage 2: Systolic Blood Pressure > 139 mmHg Diastolic Blood Pressure > 89 mmHg	Severe elevation in blood pressure levels requiring pharmacological treatment in addition to lifestyle modifications to reduce the risk of cardiovascular events

Note: Reference values reported in the table were taken from Whelton PK, Carey RM, Aronow WS, et al. 2017 ACC/AHA/AAPA/ABC/ACPM /AGS/APhA/ASH/ASPC/NMA/PCNA Guideline for the prevention, detection, evaluation, and management of high blood pressure in adults. J Am Coll Cardiol. 2018; 71(19):e127–e248.

Hypertension is highly modifiable and can be influenced by various lifestyle factors, including physical activity. Immediately after a single bout of exercise, substantial changes occur in the regulation of arterial blood pressure. This phenomenon, known as Post-Exercise Hypotension (PEH) [7, 8], is considered a relevant non-pharmacological strategy to counteract hypertension risk. Its clinical and preventive relevance lies in its ability to reproduce, after a single exercise session, the chronic reduction in blood pressure usually achieved with pharmacological treatment. PEH typically lasts about 2 hours in healthy individuals and may persist for more than 12 hours in hypertensive patients, representing a valuable preventive mechanism when exercise is performed regularly. Moreover, the magnitude in adults. J Am Coll Cardiol. 2018; 71(19):e127–e248. and duration of PEH are strongly influenced by exercise characteristics, particularly session duration, intensity, and frequency. Longer or repeated sessions are associated with more pronounced and sustained blood pressure reductions, highlighting the preventive and therapeutic relevance of structured physical activity in hypertension management [9, 10]. PEH has been attributed to several mechanisms, including vasodilation, reduced sympathetic nervous activity, and improved baroreceptor sensitivity. It is commonly associated with indoor exercise (e.g., resistance training, circuit training, and aerobic workouts), which has been shown to significantly reduce blood pressure immediately following the session [8, 11, 12]. Although indoor exercise is a popular form of physical activity, there has been a growing trend toward outdoor activities, particularly for mental health benefits, given their high salutogenic potential. Salutogenesis refers to the capacity to promote overall health and wellbeing by enhancing resilience, improving stress management, and fostering positive physical and psychological outcomes through engagement with natural environments [13, 14]. Urban green and outdoor environments are the most commonly explored and may provide benefits such as varied terrain, fresh air, and exposure to nature. These factors can enhance the overall exercise experience and amplify health outcomes [15, 16]. In particular, outdoor activities, such as hiking, offer additional benefits beyond physical exercise, including exposure to nature, accessibility for people of different abilities, minimal equipment requirements, cost-effectiveness, and the possibility for individuals to select terrain difficulty and the walking pace. Hiking, defined as walking a substantial distance in the outdoors, often over natural terrain with obstacles such as rocks and tree roots, has gained popularity in recent years [15–17]. While the increasing popularity of outdoor activities is well documented [13], less is known about their potential beneficial effects on blood pressure compared with indoor exercise. While indoor exercise is well established in inducing PEH, outdoor exercise also holds promise for achieving similar cardiovascular benefits. The choice between indoor and outdoor exercise can therefore be tailored to individual preferences and circumstances, ensuring that the benefits of PEH remain widely accessible. Considering the numerous health benefits of outdoor activities and their potential to enhance hemodynamic responses, exploring outdoor exercise as an alternative to traditional indoor exercise sessions is relevant. Therefore, this study aims to investigate blood pressure responses and PEH following outdoor exercise compared with indoor exercise.

## MATERIALS AND METHODS

Participants were recruited by means of flyers, posters, brochures, advertisements on social networks, and word-of-mouth. In line with the literature [18] the inclusion criteria were the absence of the following conditions: the presence or a known history of neuromuscular disorders, uncontrolled heart failure or hypertension, multiple sclerosis, significant cognitive impairment, acute rheumatoid arthritis, cardiovascular diseases, use of antihypertensive medications, pulmonary dysfunction, uncontrolled metabolic diseases such as diabetes, a prior diagnosis of osteoporosis, a diagnosis of obesity (Body Mass Index [BMI] ≥ 29 kg/m^2^) or any injury sustained within the past six months.

**FIG. 1:**
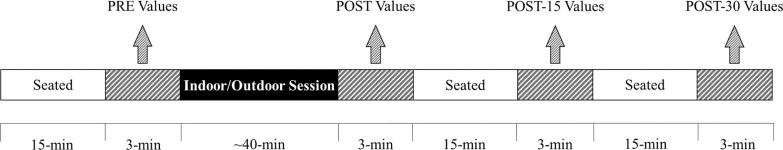
Timeline of the measurements.

Forty participants were included in the study, approved on 14 April 2022 by the Institutional Review Board of the Department of Human Sciences, Society, and Health of the University of Cassino and Lazio Meridionale (approval number 6663) in accordance with the Declaration of Helsinki. Each participant was assigned a unique **FIG. 1.** Timeline of the measurements. identification code to ensure anonymity, and the data were used exclusively for statistical purposes. Considering that physical activity positively influences cardiometabolic parameters [19], to avoid the influence of physical inactivity, participants were included only if they reported being recreationally active according to the International Physical Activity Questionnaire (IPAQ) [20] and possessed medical clearance to exercise. Since previous research [19] reported a correlation between physical activity level and BMI, and in accordance with the American College of Sports Medicine guidelines for the clinical classification of participants [2], anthropometric measurements were collected one week prior to the data collection. Body weight and height were measured using a scale and stadiometer accurate to 0.1 kg and 0.1 cm (Seca, model 709, Vogel & Halke, Hamburg, Germany), and BMI was calculated. Theoretical maximum heart rate (HRmax) was calculated for each participant, according to the equation: 220 – age (years). After one week, participants performed two different testing procedures, including one hiking session and one indoor test on a treadmill, with a minimum of 48 h recovery in between. At the start of each session, weather data were collected to ensure the reliability and accuracy of the study’s findings. To ensure consistency and confirm the absence of significant environmental differences, temperature (indoor: 24.6 ± 2.8°C, outdoor: 24.2 ± 1.8°C), humidity (indoor: 41.1 ± 4.1 %, outdoor: 42.1 ± 1.8 %) and barometric pressure (indoor: 1012.0 ± 5.1 hPa, outdoor: 1008.1 ± 4.9 hPa) levels were recorded during both sessions.

Of the 40 participants who began the study, 3 withdrew due to personal reasons. Subsequently 37 participants (18 females, age: 24.7 ± 0.7 years; body mass: 56.3 ± 9.1 kg; body height: 163.4 ± 8.9 cm; BMI: 21.1 ± 2.1 kg/m^2^; 19 males, age: 24.9 ± 0.5 years; body mass: 75.5 ± 11.9 kg; body height: 176.5 ± 8.4 cm; BMI: 24.1 ± 2.6 kg/m^2^) completed all testing and data were used for the final analysis.

The hiking session took place outdoors in Gaeta, Italy. The location was selected based on the similarity (in terms of duration and mean slope) with the indoor session and determined according to the walkability principle [21]. This principle considers physical-environmental and historical-cultural aspects of the route, such as: the presence of necessary services within walking distance, the attractiveness of the route in terms of architecture and social context, and the level of comfort and safety of the route. The identification of the hike included an evaluation of various parameters such as duration, length, slope, and energy demand as per the Club Alpino Italiano Manual. During the outdoor session, each participant completed the same hike at a self-selected speed.

During the indoor session, all participants performed a maximal incremental walking test on a treadmill (RunRace HC1200, Technogym, Cesena, Italy), incorporating both uphill and downhill walking. Participants were asked to self-select their speed [18, 22], trying to replicate their pace during the outdoor session. The initial speed was chosen to match a comfortable walking pace during the first 2-min stage, with the slope set at 0% followed by 2% slope increments every 2-min stage. When participants reached the maximum exertion, the uphill phase finished and the downhill phase started with 2% slope decrements every stage, until returning to 0% grade.

During both sessions baseline systolic and diastolic blood pressures were measured using a digital sphygmomanometer (Erkameter 3000, Erka, Bad Tolz, Germany). Resting blood pressure values were obtained 15 minutes before the start of each session (PRE), after a 15-min period of seated rest on a chair with armrests. Blood pressure was also measured immediately after the conclusion (POST), 15 minutes (POST-15) and 30 minutes (POST-30) after the sessions, to assess acute cardiometabolic responses, as shown in the measurement timeline (Figure 1).

Two measurements were recorded at each time point, and if there was more than a 5-mmHg difference between them, an additional measurement was taken. Within each measurement period, the maximum (highest reading) and minimum (lowest reading) value of systolic blood pressure, diastolic blood pressure and heart rate were collected, and average values of measurements were calculated and used for statistical purposes. PEH was calculated for systolic blood pressure as PRE – POST-30 (mmHg). Rate pressure product was calculated as systolic blood pressure (mmHg) multiplied by heart rate (bpm).

Before and during the sessions, a Garmin HRM-Pro™ (Garmin International, Kansas City, MO, USA) chest strap monitor, positioned just below the xiphoid process, was used to record real-time (at a sampling rate of 1 Hz) heart rate data and track the coordinates of the hiking route. Resting heart rate data were collected 15 minutes before each session, after a 5-min seated rest. Spatiotemporal data were recorded using a GPS Smartwatch (Garmin Forerunner^®^ 245 Music) worn on the left wrist, which was used to display real-time heart rate acquired from the Garmin HRM-Pro™ chest strap monitor.

Statistical analysis was conducted using STATA statistical software version 18 (Stata-Corp, College Station, LLC, Texas, USA). All values are reported as means and standard deviation (± SD) and the average values across measurements were used for the final analysis. Repeated-measures mixed models were used to evaluate the hemodynamic responses during and after the maximal incremental walking test on a treadmill and the hiking session and to compare with the baseline values for each test. In the model, subjects were considered as random effects, whereas the environment (indoor vs. outdoor) and testing time (PRE, POST, POST-15 and POST-30) were treated as fixed effects. Subsequently, the contrast method was employed to test whether the means of the dependent variables (i.e., systolic and diastolic blood pressure, heart rate, rate pressure product) for setting (indoor vs. outdoor) and time point (PRE, POST, POST-15 and POST-30) were equivalent. When significant main effects and interactions were found, post hoc analysis was applied using a Bonferroni correction. To avoid type 1 error, after Bonferroni correction, for the linear repeated-measures mixed model, significance was set at p < 0.05 for the main effects and at p < 0.0017 for post hoc pairwise comparisons. Cohen’s d (*d*) was used to evaluate the power of all the primary comparisons. Paired t-tests were used for comparisons between the maximal incremental walking test and the hiking session.

## RESULTS

**FIG. 2:**
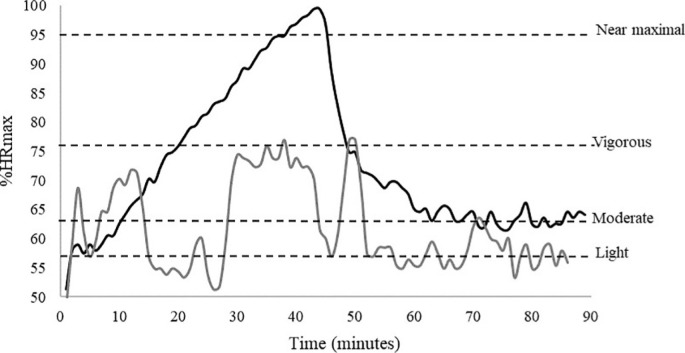
Percentage of heart rate max during indoor (black line) and outdoor (grey line) session with horizontal dashed lines identifying threshold of exercise intensities in a representative participant.

The outdoor session had a length of ~3800 m with the maximum slope at 19% and the mean slope at 5.3%. During the session participants achieved on average 84% of their theoretical HRmax over a mean duration of 40 minutes. For the indoor session the length was ~2800 m, the maximum slope provided from the treadmill was 25% and the mean slope 8.1%. During the session participants reached on average 92% of their theoretical HRmax in 41.86 minutes of mean duration. Heart rate from a representative participant during the sessions is shown in Figure 2.

Means and standard deviations of the average values and the rate pressure product during outdoor and indoor sessions are shown in Table 2.

**TABLE 2:** Blood pressure and heart rate response before, during and after outdoor and indoor sessions.

**Outdoor Session**	**PRE**	**POST**	**POST-15**	**POST-30**
Average sBP (mmHg)	120 ± 11	118 ± 8	112 ± 9^*,#^	110 ± 9^*,#^
Average dBP (mmHg)	77 ± 7	76 ± 8	73 ± 7	73 ± 8^*^
Average HR (beats · min^−1^)	67 ± 9	81 ± 12^*^	73 ± 10^*,#^	72 ± 10^#^
RPP (mmHg · bpm)	8122 ± 1381	9674 ± 1531^*^	8257 ± 1262^#^	7981 ± 1179^#^

**Indoor Session**	**PRE**	**POST**	**POST-15**	**POST-30**
Average sBP (mmHg)	120 ± 11	112 ± 10^*^	109 ± 9^*^	109 ± 9^*^
Average dBP (mmHg)	75 ± 7	73 ± 7	69 ± 6^*^	70 ± 7^*^
Average HR (beats · min^−1^)	70 ± 11	89 ± 13^*^	80 ± 12^*,#^	79 ± 11^*,#^
RPP (mmHg · bpm)	8518 ± 1537	9911 ± 1626^*^	8881 ± 1255^#^	8480 ± 1229^#^

Note: Values are mean ± SD. sBP, systolic blood pressure; dBP, diastolic blood pressure; HR, heart rate; RPP, rate pressure product.

* significantly (p < 0.0001) different from PRE values,

# significantly (p < 0.0001) different from POST values.

**FIG. 3:**
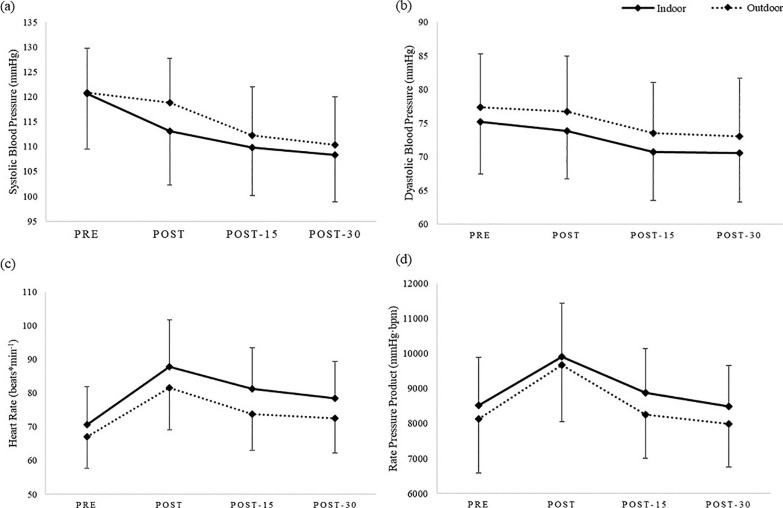
Means and standard deviations of (a) systolic blood pressure, (b) diastolic blood pressure, (c) heart rate and (d) rate pressure product between indoor session (solid lines) and outdoor session (dotted lines). All reported differences were statistically significant (p < 0.0001). (a) For the indoor session, PRE values were higher than POST, POST-15, and POST-30. For the outdoor session, POST-15 and POST-30 values were lower than PRE and POST. (b) For the indoor session, PRE was higher than POST-15 and POST-30. For the outdoor session, POST-30 was lower than PRE. (c) For the indoor session, PRE was higher than POST, POST-15, and POST-30. For the outdoor session, PRE was lower than POST and POST-15, and POST was higher than POST-15 and POST-30. (d) For both indoor and outdoor sessions POST values were higher than PRE, POST-15, and POST-30.

The repeated-measures mixed models showed a significance of model (F_(7,252)_ = 25.32; p < 0.0001) for systolic blood pressure. In particular, significant differences were found for time (F_(3,216)_ = 26.34; p < 0.0001) with PRE values higher than POST (*d* = 0.68), POST-15 (*d* = 1.04) and POST-30 (*d* = 1.19) for indoor session. For outdoor session (Figure 3a), POST-15 was significantly lower than PRE (*d* = 0.91) and POST (*d* = 0.71), and POST-30 was significantly lower than PRE (*d* = 1.12) and POST (*d* = 0.91). A significant interaction between time and setting (F_(3,216)_ = 62.34; p < 0.02) was found, although no significant differences emerged for setting (F_(1,72)_ = 1.71; p = 0.19).

Regarding diastolic blood pressure, a main effect was found for time (F_(3,216)_ = 20.72; p < 0.0001), with PRE values significantly higher than POST-15 (*d* = 0.59) and POST-30 (*d* = 0.61) in the indoor session. For the outdoor session (Figure 3b) POST-30 values were significantly lower than PRE (*d* = 0.52). No significant difference emerged for setting (F_(1,72)_ = 2.69; p = 0.1) and no interaction was found between time and setting (F_(3,216)_ = 0.10; p = 0.95).

The overall model was statistically significant (p < 0.0001) for heart rate, with a difference for setting (F_(1,72)_ = 5.85; p = 0.01) and time (F_(3,216)_ = 94.33; p < 0.0001). PRE values were significantly lower than POST (*d* = 1.36), POST-15 (*d* = 0.90) and POST-30 (*d* = 0.71) for indoor session. For outdoor session (Figure 3c) PRE values were significantly lower than POST (*d* = 1.31) and POST-15 (*d* = 0.66) and POST values were significantly higher the POST-15 (*d* = 0.66) and POST-30 (*d* = 0.78). Regarding time and setting, no significant interactions were found (F_(3,216)_ = 1.49; p = 0.21).

The overall model for rate pressure product was statistically significant (F_(7,252)_ = 23.87; p < 0.0001), with a significant effect of time (F_(3,216)_ = 61.52; p < 0.0001). POST values were significantly higher than PRE (indoor *d* = 0.87, outdoor *d* = 1.06), POST-15 (indoor *d* = 0.71, outdoor *d* = 1.01) and POST-30 (indoor *d* = 0.99, outdoor *d* = 1.23) for both sessions (Figure 3d). No significant differences were found for setting (F_(1,72)_ = 2.47; p = 0.12) and no interaction between time and settings (F_(3,216)_ = 0.78; p = 0.51). Lastly, paired t-test showed no significant difference between settings for PEH (p = 0.36).

## DISCUSSION

This study aimed to investigate the effect of a hiking session on blood pressure and PEH compared with an indoor session of approximately the same intensity and duration. The main findings indicated that large hemodynamic changes occur during both maximal walking test and hiking session in recreationally active participants. Regardless the proposed activities, systolic blood pressure, diastolic blood pressure and heart rate showed significant decreases over time, with lowest values observed at POST-30. These results are consistent with previous research [23], confirming the benefits of the exercise on acute reduction of blood pressure and offering a new perspective for investigation. While most of the literature focuses on inactive individuals, who typically present with higher blood pressure and a greater reduction in blood pressure after exercise, the present study found that recreationally active adults also experienced considerable decreases, reinforcing the value of exercise for blood pressure control [23, 24]. The lack of differences in blood pressure responses between the two settings, despite variations in internal load (%HRmax reached during the sessions), highlights the potential of hiking for cardiovascular benefits and emphasizes the generalizability of the PEH effect.

The systolic blood pressure showed a significant reduction over time, with a higher reduction in POST-30. The analysis also revealed a significant interaction between the time points and exercise settings, indicating different hemodynamic responses based on the stimulus intensity. Specifically, the systolic blood pressure had two different trends during the sessions, with a greater reduction between POST-15 and POST-30 in the hiking session, and between POST and POST-15 in the indoor session. These results can be explained by the different intensities of two exercises. Since the indoor session was a maximal incremental walking test, the reached intensity, in terms of % of HRmax, was higher than outdoor session. Conversely, during the outdoor session, the increment of the workload was not as regular as the indoor one (every 2-min stage). Examining the effects of high-intensity training, the literature [25] reported significant improvements in systolic blood pressure, with notable reductions immediately post-exercise, indicating a pronounced cardiovascular response. Moreover, comparing the effects of low, moderate, and vigorous intensity exercise sessions among normotensive young adults higher exercise intensities have been found to elicit the largest acute reductions in blood pressure [26]. However, in the present study, during POST-30, both sessions exhibited lower systolic blood pressure values than at rest, confirming the positive effect of exercise on the management of blood pressure.

For diastolic blood pressure, the trends between sessions were largely similar. A significant reduction was observed over time, particularly between PRE and POST-30 (p < 0.001) in both sessions. This findings align with existing literature, which reports smaller but more consistent reductions in diastolic compared with systolic blood pressure after exercise.

Aerobic exercise has been shown to lower diastolic blood pressure by a few mmHg, reflecting beneficial effects on cardiovascular health [27]. This reduction is primarily attributed to the vasodilatory effects of exercise, which decrease vascular resistance and improve arterial compliance [28]. These results highlight the preventive role of PEH in healthy young populations. Although participants in this study were recreationally active young adults, elevated blood pressure during youth and early adulthood has been associated with an increased risk of vascular alterations and progression to hypertension later in life [29, 30]. Moreover, PEH reflects vascular adaptations such as improved arterial compliance, enhanced endothelial function, and reduced peripheral resistance [7, 8]. Demonstrating that this effect also occurs in healthy young individuals reinforces the preventive role of regular physical activity in promoting cardiovascular health across the lifespan. This suggests that PEH may serve as an early preventive mechanism, even in healthy populations, by counteracting the gradual vascular changes that can lead to hypertension later in life.

Heart rate recovery after exercise reflected significant time effects, explained by increasing parasympathetic activity leading to a return to resting values [31]. Differences in heart rate between settings, similar to those observed for systolic blood pressure, can be attributed to session intensity.

For rate pressure product, the significant time effect reflected the natural recovery process of the cardiovascular system, with values significantly decreasing from immediately after the end of the sessions to 30 minutes post-exercise. Rate pressure product is a noninvasive predictor of myocardial workload, as it is directly related to myocardical oxygen consumption [32, 33]. No differences were found between outdoor and indoor sessions, suggesting that both exercises could have a similar myocardial workload. Therefore, the similarity in rate pressure product across both sessions suggests that exercising outdoors could be a valid alternative to indoor aerobic exercise (e.g., on treadmill), taking the advantages of outdoor activities (high enjoyment, air quality, social relationships).

The findings suggested that outdoor hiking session and indoor walking test showed similar cardiovascular responses, given the absence of statistic differences between both sessions in blood pressure (systolic and diastolic) values and for rate pressure product. These results are further supported by the PEH findings, indicating that both sessions effectively reduce blood pressure after exercise with similar magnitudes of PEH. The similar reductions in post-exercise suggest that individuals can achieve effective cardiovascular responses and benefits from either exercise modality. Those who prefer outdoor activities like hiking can expect similar improvements in cardiovascular health as those who engage in structured indoor exercises. Given that outdoor activities are often perceived as more enjoyable [34], opportunity to promote them may provide a sustainable and engaging way to replicate the benefits of traditional physical activity while further supporting cardiovascular health. The novelty of this study lies in directly comparing indoor and outdoor walkingbased exercise in recreationally active young adults, a group less frequently examined in PEH research. Our results demonstrate that significant reductions in systolic and diastolic blood pressure also occur in this population, reinforcing the preventive role of exercise. Moreover, the comparison between an indoor maximal incremental walking test and an outdoor hiking session provides evidence that PEH is not limited to laboratory-based protocols but also extends to realworld outdoor settings. This expands the generalizability of PEH and underlines the potential of enjoyable, low-cost activities, such as hiking, to promote cardiovascular health.

## CONCLUSIONS

Findings from this study suggest that while significant variations in physiological parameters over time occur, the environment (indoor vs. outdoor) does not significantly influence these changes. Both indoor treadmill and outdoor hiking sessions induced a clear postexercise hypotensive effect lasting up to 30 minutes after exercise, confirming the relevance of PEH as a short-term protective mechanism against hypertension. These findings align with existing literature and confirm the positive effect of physical activity on reducing blood pressure [3, 35]. From a practical perspective, our results suggest that young, recreationally active individuals can achieve similar cardiovascular benefits regardless of whether exercise is performed indoors or outdoors. However, outdoor activities may offer additional advantages, such as lower perceived exertion and greater long-term adherence, which could further support cardiovascular health. This enhancement is linked to the lower internal load observed during outdoor activities, suggesting a protective cardiovascular effect, even with similar exercise characteristics. Outdoor activities replicate traditional physical activity while offering a potentially more sustainable and beneficial approach to improving overall health by reducing cardiovascular stress. Practically, this indicates that a single 40-min walking session, whether indoors or outdoors, is sufficient to induce a clinically relevant short-term reduction in blood pressure, reinforcing the value of integrating regular exercise into daily routines. Therefore, incorporating outdoor exercise sessions, when feasible, represents a sustainable and accessible strategy to promote regular physical activity and reinforce the protective effects of PEH in daily life.

### Limitations

Several limitations should be considered when interpreting these findings. The observation window was limited to 30 minutes postexercise, preventing conclusions regarding the longer-term persistence of PEH. The assessment did not include measures of subjective enjoyment or affective responses, which might have provided additional insight into the broader impact of indoor and outdoor exercise. The sample consisted exclusively of young, recreationally active adults, limiting the generalizability of the results to other populations, such as sedentary individuals, older adults, or those with hypertension. In addition, although temperature and humidity were controlled to minimize environmental variability, other contextual factors, such as noise, light exposure, or social interactions, were not systematically monitored and may have influenced cardiovascular responses.

### Future Research Directions

Future research should address these limitations and expand the current findings in several directions. Longer follow-up periods are warranted to determine the persistence of PEH and to explore potential differences in recovery between exercise modalities. Investigating the role of subjective enjoyment, psychological well-being, and adherence in response to indoor versus outdoor activities could provide valuable insights into long-term exercise adoption. Additionally, studies should include more diverse populations, such as older adults, individuals with hypertension, or clinical groups, to enhance external validity. Future investigations might also examine environmental and seasonal influences, such as thermal stress, solar radiation, or air quality, to better understand how outdoor conditions affect cardiovascular and perceptual responses to exercise. Finally, comparing different types of outdoor environments (e.g., urban vs. natural settings) may help clarify the specific contribution of nature exposure to cardiovascular health. Such investigations would build on the current findings by clarifying how contextual and environmental factors modulate the magnitude and persistence of PEH.
